# Supplementary material for: A Novel Role for the Tumor Suppressor Gene ITF2 in Tumorigenesis and Chemotherapy Response
Source: Cancers (Basel). 2020 Mar 26;12(4):786. doi: 10.3390/cancers12040786 (PMC7226299; doi:10.3390/cancers12040786)
Supplement: Supplementary file 1 [file cancers-12-00786-s001.pdf]

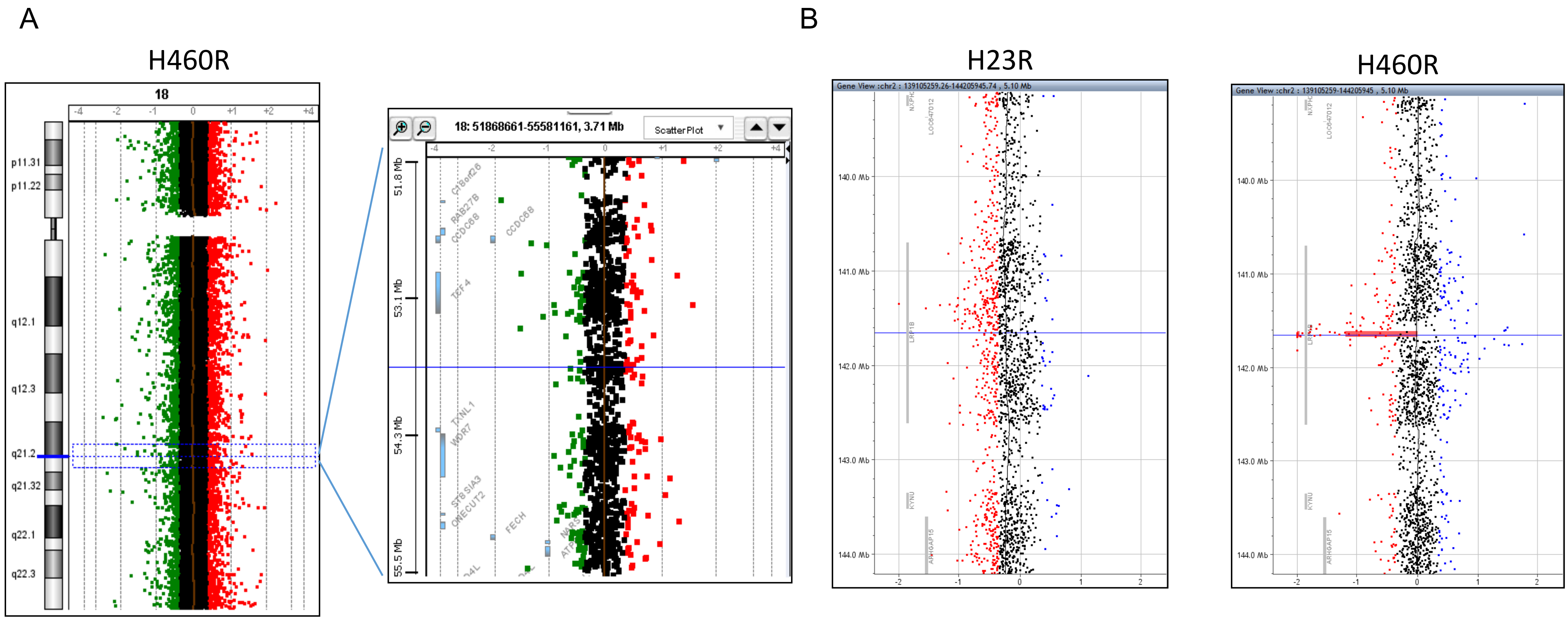

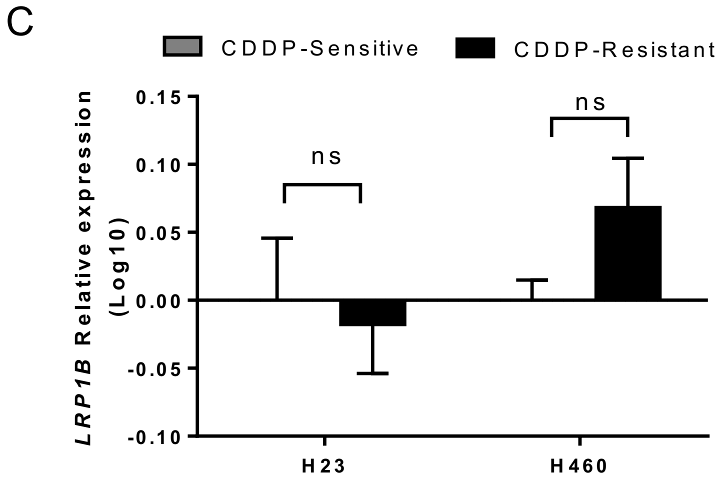

Supplementary Figure 1: (A) Picture extracted from the Agilent Cytogenomics 3.0.1.1 software showing the absence of ITF2 deletion in the H460R cells. (B) Picture extracted from the Agilent Cytogenomics 3.0.1.1 software showing the *LRP1B* deletion in chromosome 2 in H23R and H460R cell lines. (C) Relative mRNA expression levels of *LRP1B* measured by qRT-PCR. The results show the mean fold induction compared to the sensitive cells. Gene expression was normalized to *GAPDH*. Data represent the relative expression levels obtained from the combination of two independent experiments measured in triplicate ± SD. ns: not significant.

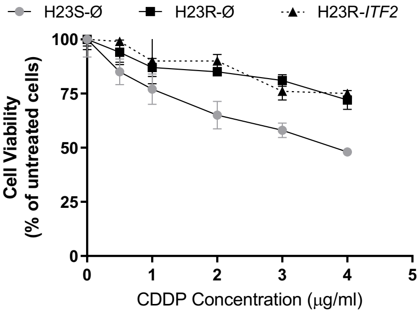

Supplementary Figure 2: Effect of *ITF2* on cisplatin resistance in H23 cells. (A) Viability curves of H23 cell lines transfected with pCMV6 (S-Ø and R-Ø) and with the overexpression vector (R-ITF2). Each experimental group was exposed to six different CDDP concentrations for 48h. Data were normalized to each untreated control, set to 100%. The data represent the mean ± SD of at least three independent experiments performed in quadruplicate at each drug concentration for each cell line analyzed.

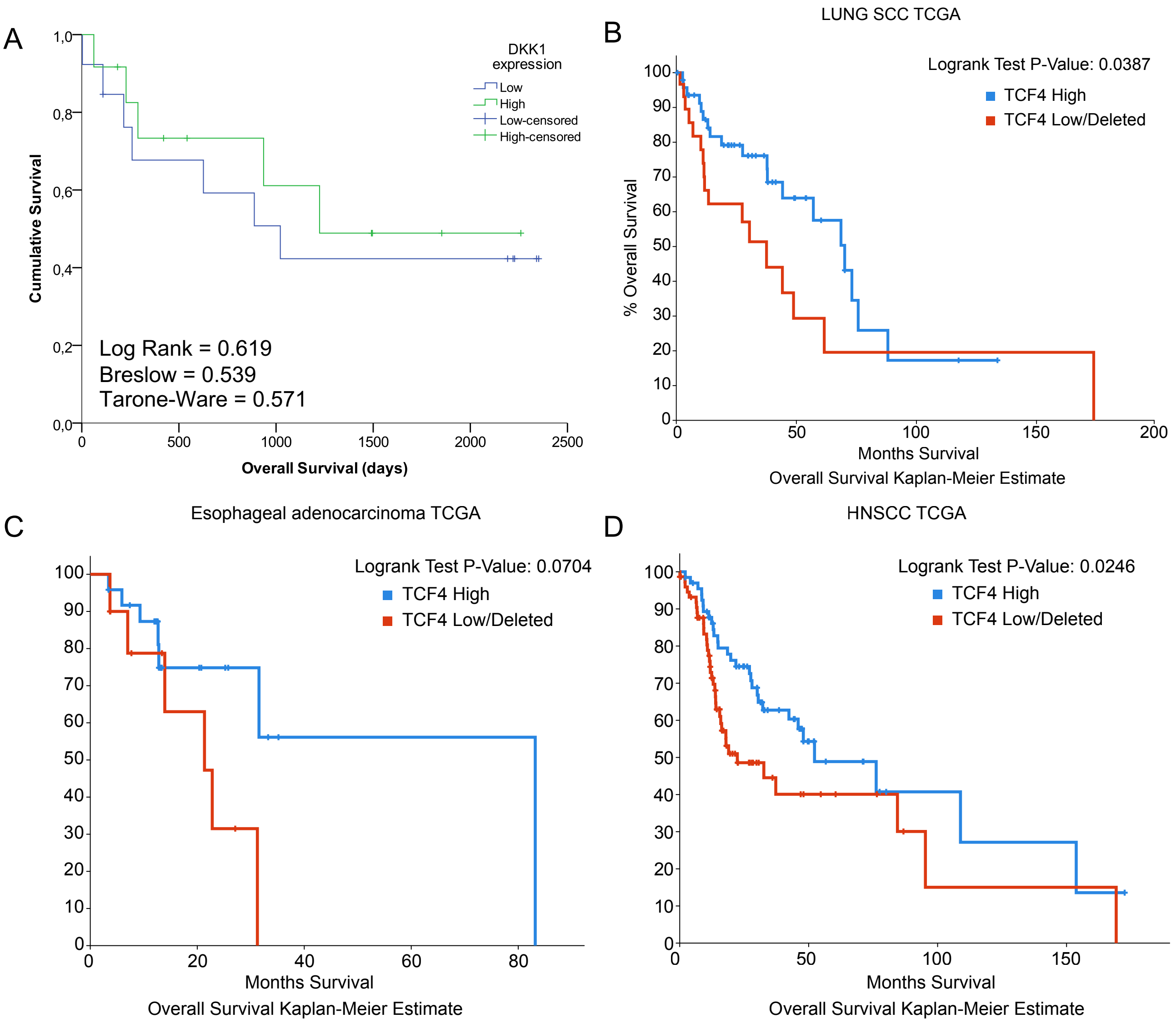

Supplementary Figure 3: (A) Kaplan-Meier analysis in a cohort of 25 NSCLC patients from Hospital la Paz for *DKK1* expression. LogRank, Breslow and Tarone-Ware tests were used for comparisons and $p<0.05$ was considered as a significant change in OS. (B-D) Kaplan-Meier analysis on TCGA datasets from lung SCC (n=230; High n=48; Low/Del n=33)(B), esophageal adenocarcinoma (n=186: High n=25; Low/Del n=41)(C) and head and neck SCC (n=522; High n=66; Low/Del n=74)(D) comparing tumors with deletion or low expression of *TCF4* compared with tumor with increased *TCF4* expression.

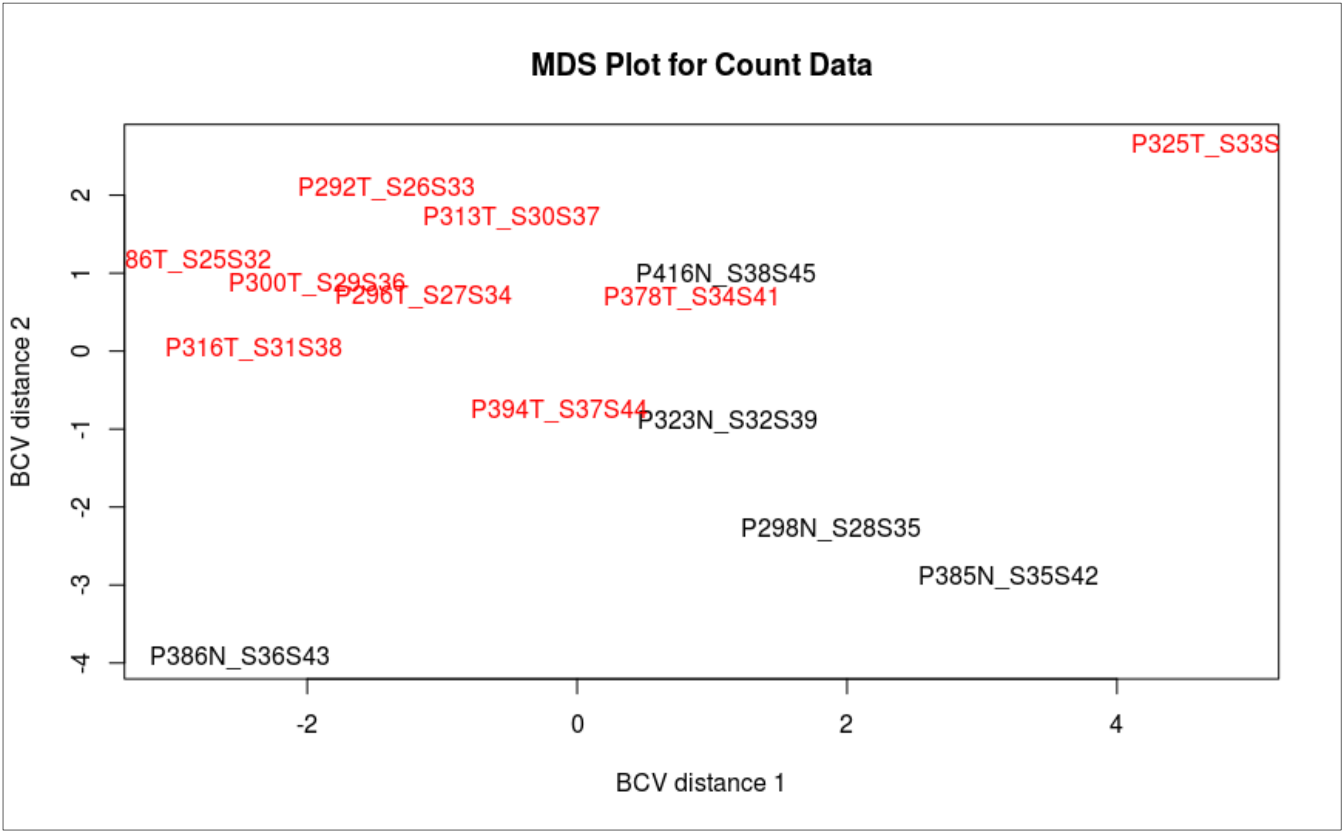

Supplementary Figure 4. Plot samples on a two-dimensional scatterplot. In this plot, distances are based on biological coefficient of variation (BCV, square root of the common dispersion). A set of the 500 most differentially expressed genes with the largest biological variation among the libraries were chosen for the analysis. Samples P386 (LC1) and P385 (LC2) are lung tissue samples of non-neoplastic origin from autopsies. Samples P298 (Pat 9), P323 (Pat21) and P416 (Pat 25) are adjacent normal tissue (ATT) from NSCLC patients.

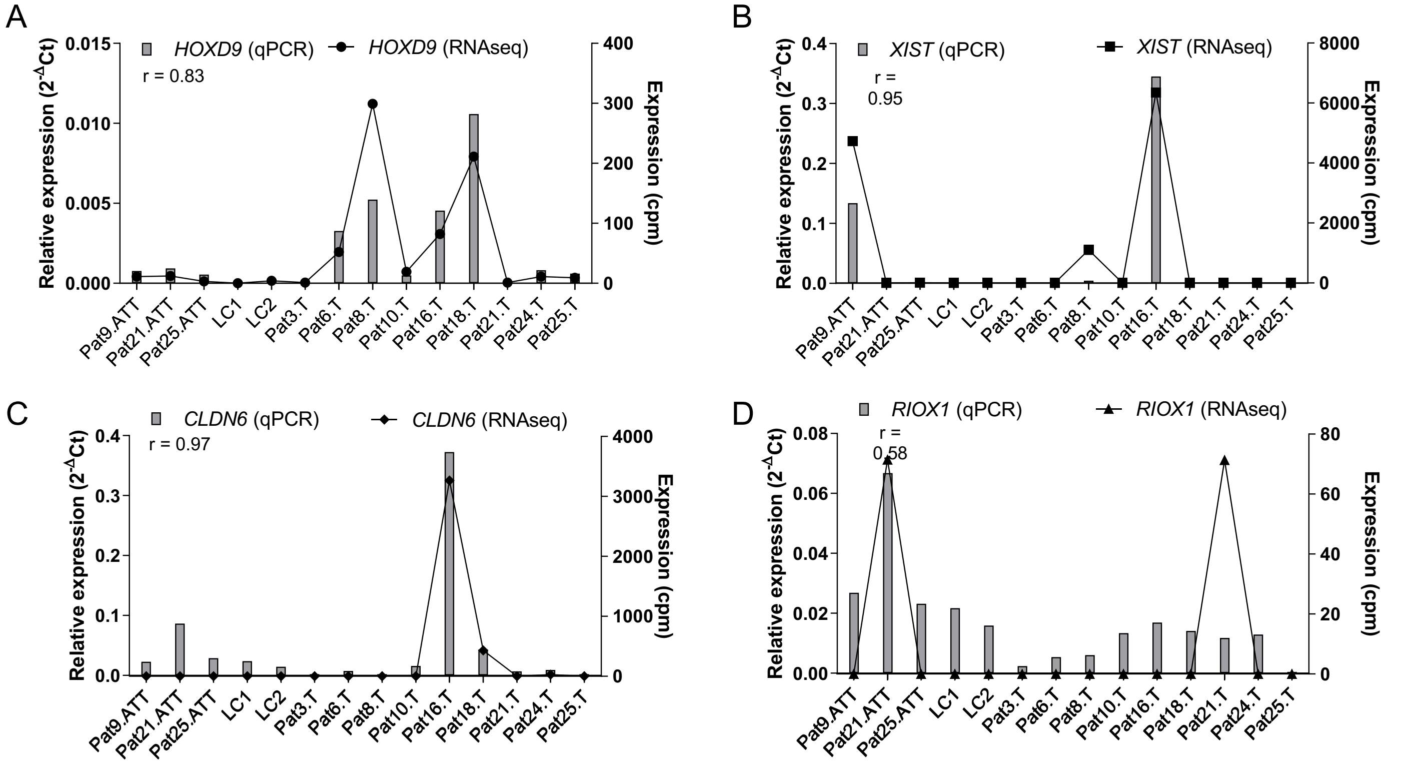

Supplementary Figure 5. Expression levels of candidate target genes measured by RNA-seq and qRT-PCR in tumor and non-tumor samples from NSCLC patients. (A-D) correlation between RNA-seq and qRT-PCR expression levels performed in a selection of 14 samples in *HOXD9* (A), *XIST* (B), *CLDN6* (C) and *RIOX1* (D). Bars represent the relative expression of each gene measured by qRT-PCR in triplicate and represented as $2^{-\Delta Ct}$ and lines represent the count per million obtained from the RNA-seq analysis.

Supplementary Table 1. Summary of the common deleted regions and genes in resistant cells. bp (base pairs)

Chromosome	Cytoband	H23/H460<br>Deleted region	A2780/OVCAR3<br>Deleted region	H23/A2780/OVCAR3<br>Deleted region	Common Nº bp	Common Genes
chr2	q22.1	141618089-141673466			55377	*LRP1B*
chr9	q22.33		99315964-99362235		46271	*TMOD1*
chr18	q21.2 - q21.31			50304876-53014765	2709889	*C18orf26, RAB27B, CCDC68, TCF4, TXNL1, WDR7, BOD1P*
chr18	q21.32			54661447-55069272	407825	*ZNF532, LOC390858, SEC11C, GRP*

Supplementary Table 2. Clinicopathological and experimental data obtained from patients with ovarian cancer from La Paz University Hospital

Patient	Histology	Stage	Grade	Chemotherapy	Status	OS (days)	*ITF2* ($2^{-\Delta\Delta Ct}$)	*DKK1* ($2^{-\Delta\Delta Ct}$)
1T	Serous	IIIC	Differentiated	CBDCA+Paclitaxel	Alive	813	0.102	2.32
2T	Serous	NA	Differentiated	No	Exitus	1060	0.113	0.41
3T	Serous	NA	Poorly differentiated	No	Exitus	14	0.045	0.033
4T	Serous	IIC	Poorly differentiated	CBDCA+Paclitaxel	Alive	1632	0.02	0.041
5T	Endometrioid	IA	Differentiated	No	Alive	1736	0.048	0.09
6T	Serous	III	Poorly differentiated	CDDP+Paclitaxel	Alive	1013	0.023	0.056
7T	Serous	IV	Poorly differentiated	CBDCA+Paclitaxel	Exitus	498	0.041	0.045
8T	Clear Cell	III	NA	CBDCA+Paclitaxel	Exitus	1135	0.059	0.231
9T	Serous	IIIC	Poorly differentiated	CBDCA+Paclitaxel	Exitus	716	0.122	0.354

Note: OS, Overall Survival; CDDP: cisplatin; CBDCA: carboplatin; NA: not available.

Supplementary Table 3. Clinicopathological and experimental data of *ITF2, DKK1, CDLN6* and *XIST* for the NSLC patients that were used for RNAseq

Patient	Histology	Sex	Stage	Chemotherapy	Relapse	Status	OS (days)	PFS (days)	*ITF2* ($2^{-\Delta Ct}$)	*DKK1* ($2^{-\Delta Ct}$)	*CLND6* ($2^{-\Delta Ct}$)	*XIST* ($2^{-\Delta Ct}$)	*RIOX1* ($2^{-\Delta Ct}$)
Pat3.T	Epidermoid	Male	IB	No	Yes	Exitus	1022	825	0.27	1.25	1.18·10-3	2.71·10-7	2.33·10-3
Pat6.T	Large cell	Male	IIB	No	No	Exitus	62	62	0.43	1.97	7.43·10-3	3.92·10-6	5.39·10-3
Pat8.T	Epidermoid	Female	IIIB	CDDP + Others	No	Exitus	109	109	0.18	0.43	3.63·10-4	4.16·10-3	6.03·10-3
Pat10.T	Epidermoid	Male	IB	No	No	Alive	1853	1853	0.42	2.78	1.58·10-2	7.09·10-3	1.43·10-2
Pat16.T	Adenocarcinoma	Female	IIIA	CDDP + Others	ND	Alive	2228	2228	0.24	0.08	3.73·10-1	3.45·10-1	1.69·10-2
Pat18.T	Epidermoid	Male	IIB	CBDCA + Others	No	Exitus	259	259	0.15	0.03	4.31·10-2	2.67·10-4	1.41·10-2
Pat21.T	Adenocarcinoma	Male	IIIA	CDDP + Others	No	ND	421	421	0.20	4.72	6.92·10-3	4.32·10-4	1.18·10-2
Pat24.T	Adenocarcinoma	Male	IIA	CBDCA + Others	No	Alive	1491	1491	0.98	10.34	9.09·10-3	3.64·10-4	1.29·10-2
Pat25.T	Adenocarcinoma	Male	IIA	CDDP + Others	No	Alive	1496	1496	0.77	137.16	5.91·10-7	1.93·10-5	1.70·10-5

Supplementary Table 4. Selected genes potentially involved in the Wnt signaling pathway identified through a global transcriptomic analysis on 14 samples of NSCLC patients

**Candidates**	**Contrast A**		**Contrast B**		**Contrast C**		**Statistical Significance** 1=FDR<0.05; 0=FDR>0.05		
Gene ID	logFC	FDR	logFC	FDR	logFC	FDR	A	B	C
HOXB8	4.529	0.047	-4.644	0.035	1.352	0.547	1	1	0
HOXD9	3.615	0.042	-3.723	0.027	1.317	0.518	1	1	0
CLDN6	8.822	0.033	-7.720	0.022	2.724	0.309	1	1	0
AC022596.6	7.159	0.046	-7.566	0.028	1.207	0.628	1	1	0
SLC13A2	0.921	0.833	-6.648	0.004	-4.199	0.024	0	1	1
XIST	-0.171	0.943	-11.413	4.39E-08	-10.025	0.003	0	1	1
RIOX1	-0.785	0.843	-7.632	0.016	-6.807	0.026	0	1	1
AL591684.1	-0.022	0.971	-7.147	0.022	-5.554	0.018	0	1	1
PTPN20A	-0.643	0.751	-3.996	0.043	-3.162	0.005	0	1	1

Note: logFC: Log of FoldChange, FDR: False Discovery Rate.

Supplementary Table 5. Cell Authentication. Genomics Core Facility. IIBm CSIC-UAM.

Name of Cell line	Cancer type	Testing Date												REF	Match/Not Match
Sample			*M musculus*	D5S818	D13S317	D7S820	D16S539	VWA	TH01	AMEL	TPOX	CSF1PO	D21S11		
REF NCI-H23			Negative	12,13	12	9,10	11	16,17	6	X	8,9	10		ATCC ® CRL-5800	
H23	Lung	06/22/2016	********	12,13	12	9,10	11	16,17	6	X	8,9	10	*30*		Match
REF NCI-H460			Negative	9,10	13	9,12	9	17	9.3	X,Y	8	11,12		ATCC ® HTB-177	
H460	Lung	06/22/2016	********	9,10	13	9,12	9	17	9.3	X,Y	8	11,12	*30*		Match
REF A2780			Negative	11,12	12,13	10	11,13	15,16	6	X	8,10	10,11	*27,28*	SIGMA	
A2780	Ovary	06/22/2016	********	11,12	12,13	10	11,12,13	15,16	6	X	8,10	10,11	*28*		Match
REF OVCAR3			Negative	11,12	12	10	12	17	9,9,3	X	8	11,12		ATCC ® HTB-161	
OVCAR3	Ovary	06/22/2016	********	11,12	12	10	12	17	9,9,3	X	8	11,12	*29,31,2*		Match

Method:

STR amplification kit	GenePrintR 10 System (Promega)
STR profile analysis software	GeneMapper® v3.7 (LifeTechnologies)
Genomic Analyzer System	ABI 3130 XL (Applied Biosystems)
DNA source	Cultured cells; cultured cells pellet
DNA isolation method	DNeasy blood and tissue kit (Qiagen)
DNA quantification method	Qubit 2.0 Fluorometer (Life Technologies)
Amount of DNA/amplification	4 ng

The GenePrint® 10 System allows co-amplification and three-color detection of ten human loci: TH01, TPOX, vWA, Amelogenin, CSF1PO, D16S539, D7S820, D13S317, D21S11 and D5S818. These loci collectively provide a genetic profile with a random match probability of 1 in 2.92 × 109 and are used for human cell line and tissue authentication and identification and human cell line cross-contamination determination. STRs profiles are sent for comparison with cell line date bases like ATCC (American Type Culture Collection), DSMZ (Deutsche Sammlung von Mikrorganismen and Zellkulturen).

Supplementary Table 6: List of designed primers used for RT-PCR and the amplification characteristics.

GeneSymbol	cDNA Primer Forward	cDNA Primer Reverse	cDNA Amplification Length	Annealing Temperature	Cycles of amplification
DKK1	CAACTACCAGCCGTACCCGTG	AACAGAACCTTCTTGTCCTTTG	331	59ºC	36
HOXD9	AGCAGCAACTTGACCCAAACAACC	TGACCTGTCTCTCTGTTAGGTTGAG	189	59ºC	36
CLDN6	ATCTCCTTCGCAGTGCAGCTCCTTCAAC	TGAGTCGTACACCTTGCACTGCATC	241	59ºC	36
XIST	ATTATAAGTGACCACAGCCATGCAC	TTCATGTCCAAGGTGAGTGCCTATGCTC	334	58ºC	36
RIOX1	ACCGCTGACCTGGATTCGATGCTGCG	AACGTTGGAGCCTGCCATGCTTCC	252	62ºC	34
GAPDH	GAGAGACCCTCACTGCTG	GATGGTACATGACAAGGTGC	135	58ºC	25
